# Discriminative validity of the EQ-5D-5 L and SF-12 in older adults with arthritis

**DOI:** 10.1186/s12955-019-1129-6

**Published:** 2019-04-17

**Authors:** Andrews K. Tawiah, Fatima Al Sayah, Arto Ohinmaa, Jeffrey A. Johnson

**Affiliations:** 1 Faculty of Rehabilitation Medicine, University of Alberta, 3-44 Corbett Hall, Edmonton, AB T6G 2G4 Canada; 2 School of Public Health, University of Alberta, 2-040 Li Ka Shing Center for Health Research Innovation, Edmonton, AB T6G 2E1 Canada; 3 School of Public Health, University of Alberta, 3-267 Edmonton Clinic Health Academy, Edmonton, AB T6G 1C9 Canada

**Keywords:** EQ-5D-5 L, SF-12, SF-6D, arthritis, pain, discriminative validity

## Abstract

**Background:**

The EQ-5D-5 L and the SF-12 are the most commonly used generic measures of health-related quality of life among people with arthritis. However, there is little evidence on the extent to which the individual dimensions and domains of these instruments perform among this population. The objective was to examine the discriminative validity of the EQ-5D-5 L and the SF-12 version 2 (and SF-6D) in capturing the burden of arthritis on health-related quality of life in older adults.

**Methods:**

Cross-sectional data from the Alberta Retired Teachers Association survey were used. A known-groups approach, with a-priori hypotheses, was used to examine the discriminative validity of the domain and summary scores of the EQ-5D-5 L and the SF-12 version 2 (and SF-6D). Groups were defined based on self-reported of arthritis, chronic pain level, presence and number of comorbidities, and self-reported health status.

**Results:**

Mean age of respondents (*N* = 2844) was 68.6 (standard deviation [SD] 5.9) years; 54.8% were female, with mean body mass index (BMI) of 27.2 kg/m^2^ (SD 4.8), and 36.6% reported having arthritis. The overall mean EQ-5D-5 L index score was 0.86 (SD 0.11) and that of SF-6D was 0.79 (SD 0.13). Participants with arthritis had lower EQ-5D-5 L index score (0.83, SD 0.13) and SF-6D index score (0.75, SD 0.13) compared to those without arthritis (0.88, SD 0.09 and 0.81, SD 0.12, respectively). EQ-5D-5 L and SF-6D index scores demonstrated moderate discriminative validity with a moderate effect size (0.5). Related dimensions and domains between the EQ-5D-5 L and SF-12 (e.g., mobility with physical functioning score, pain/discomfort with bodily pain and anxiety/depression with mental health) were moderately to strongly correlated (r = 0.6–0.7). Both instruments could not adequately discriminate between participants with moderate and severe chronic pain of 6-month duration.

**Conclusion:**

Overall, the EQ-5D-5 L pain/discomfort and mobility dimensions, and the SF-12 bodily pain scale had moderate discriminative ability among older adults with arthritis. However, both instruments had limited discriminative ability for chronic pain. The importance and nature of chronic pain assessment in a given application need to be considered when choosing any of these instruments for measuring health-related quality of life in this patient population.

**Electronic supplementary material:**

The online version of this article (10.1186/s12955-019-1129-6) contains supplementary material, which is available to authorized users.

## Background

Arthritis is one of the most common chronic conditions affecting men and women in Canada [1]. In 2014, around 4.8 million (16.5%) Canadians aged 15 and older reported having arthritis; this prevalence is projected to increase to 7 million (20%) by 2031 [1, 2]. The impact of arthritis on the Canadian health care economy and lost productivity was estimated to be 33 billion dollars in 2011 [3]. Osteoarthritis and rheumatoid arthritis are the most common forms of arthritis in Canada [3]. Arthritis brings with it a burden of pain and disability that affect those living with this disease every day [3]. Because of the chronic nature of arthritis, health related quality of life (HRQL) has become an important indicator of the burden of this disease [4]. Persons with arthritis report an average of 4.0 more days of impaired physical health and 2.3 more days of impaired mental health during the previous 30 days compared to those without arthritis [4].

Generic preference-based measures such as the EQ-5D [5] and health profile measures such as the SF-12 [6] have been most commonly used in assessing HRQL of arthritis patients. The ability of the EQ-5D and SF-12 [7] to discriminate between patients with different severity levels of arthritis will help determine the health burden in this population. Discriminative validity is the ability of an instrument to discriminate between different groups who would differ in their HRQL [8]. Evidence on discriminative validity can inform the choice of these measures for use in specific applications.

The EQ-5D initially had 3 levels (3 L) of problems for each of the five dimensions (mobility, self-care, usual activities, pain/discomfort, and anxiety/depression). More recently, a 5-level (5 L) version of the EQ-5D has been developed, with the aim of improving measurement of health status [9]. Several studies have compared the measurement properties of the EQ-5D-3 L and SF-36, SF-12 or SF-6D (a preference-based single index of health derived from the SF-36 or SF-12) questionnaires among patient with knee pain, osteoarthritis, rheumatoid arthritis and psoriatic arthritis [5, 10–13]. In these studies, the severity of these conditions was measured with the Western Ontario and McMaster Universities Osteoarthritis Index (WOMAC) and the Michigan Hand Questionnaire. Evidence on the discriminative validity of the instruments from these studies is inconclusive and highlights the extent of disagreement in literature on the discriminative ability of the EQ-5D-3 L and SF-12 questionnaires in arthritis [5, 10–13]. Importantly, all of these studies were conducted using the 3 L version of the EQ-5D and did not examine the discriminative validity of the dimension and domain scores of each of these instruments [5].

There is little evidence on how well can the EQ-5D-5 L discriminate between patients with arthritis, how it compares to the SF-12, and how each of the individual dimensions and scales of these measures performs in this patient population. Our objective was to examine the discriminative validity of the individual components and overall scores of the EQ-5D-5 L and the SF-12 version 2 (and the SF-6D) in older adults with arthritis.

## Methods

### Data

This study involved secondary analysis of cross-sectional data collected through a survey of members of the Alberta Retired Teachers Association (ARTA), in Alberta, Canada. The objective of the ARTA survey was to better understand the factors that affect care and health outcomes in this population of older adults. Participants were recruited by sending an email with a link to the online survey to all members registered with ARTA (*N* = 14,248). Participants were included if they were retired Alberta Teachers (as described by the Alberta Retired Teachers Association). There was no restriction on gender, race, and health status. Participants were able to understand, read, speak English and they were of sound mind to complete the survey. A total of 6275 (44%) participants responded to the survey, with 2514 (18%) completing the survey. Participant information sheet and consent were completed before participating in the survey. Ethical approval was obtained from the health research ethics board at the University of Alberta.

### Measures

The EQ-5D-5 L is a preference-based measure of HRQL [9]. It consists of five dimensions (mobility, self-care, usual activities, pain/discomfort and anxiety/depression) and a visual analogue scale (VAS). Each dimension has 5 levels of problems: 1 “no problems”, 2 “slight problems”, 3 “moderate problems”, 4 “severe problems” and 5 “extreme problems”. Respondents are asked to indicate their level of functioning on each of the five dimensions on that day [9]. The EQ-5D-5 L describes 3125 distinct health states, with 11111 representing the best and 55555 the worst possible health states. Index scores were generated using the Canadian scoring algorithm [14], ranging from − 0.148 for worst (55555) to 0.949 for best (11111) health states [14]. A minimally important difference (MID) for the index score has been suggested as 0.04 [15]. The VAS score ranges from 0 (worst health imaginable) to 100 (best health imaginable).

The SF-12 version 2 is derived from items in the SF-36 health survey [16]. It measures eight domains of functioning and well-being, asking respondents to consider their health status over the past 4 weeks: physical functioning (PF), role limitations due to physical problems (RP), bodily pain (BP), general health perceptions (GH), energy and vitality (VT), social functioning (SF), role limitations due to emotional problems (RE) and mental health (MH) [16]. The eight domains can further be summarized into physical component summary (PCS) and a mental component summary (MCS) scores, which are interpreted as standardized T-scores, with a mean of 50 and SD of 10. SF-12 scales and summary scores were calculated according to Fleishman, Selim and Kazis [14]. Domain scores were also converted into T-scores for this analysis. The SF-6D is derived using 7 items from the SF-12. The SF-6D classification system consists of six domains including PF, RP, BP, VT, SF and MH describing 7500 unique health states [6]. SF-6D index scores were generated based on the UK general population preferences, ranging from 0.345 for worst (345555) to 1.0 for best (111111) health states [6]. The first item of the SF-12 (SF-1) was also used as an indicator of the overall health of participants.

Pain intensity was measured using the 11-point Numeric Pain Rating Scale. Participants indicated their pain intensity over the past 6 months. Pain intensity was subsequently categorized into: 0-no pain, 1–3-slight pain, 4–6-moderate pain, and 7–10-severe pain [17, 18]. Comorbidities were assessed using self-reported chronic condition question. Participants were asked if a health professional has ever told them that they had any of the following 13 conditions: arthritis (e.g. osteoarthritis, rheumatoid), respiratory diseases, cardiovascular diseases, diabetes, high lipids, renal diseases, bowel disorders, musculoskeletal disorders, thyroid dysfunction, eye diseases, cancer, neurological diseases, and mental/psychological conditions. The total number of comorbidities reported by each participant was calculated, and categorized into: 0, 1, 2, and 3 or more chronic conditions. Other data included age, sex, highest level of education, current employment status, total annual household income, ethnicity, and body mass index (BMI), calculated using self-reported weight and height.

### Statistical Analysis

Participants with complete EQ-5D-5 L and SF-12 data were included in this analysis (*N* = 2844). Descriptive statistics were computed for all variables. Ceiling effects for each measure were reported as the percentage of participants in the best possible health states (11111 for EQ-5D-5 L and 111111 for SF-6D).

The discriminative validity of the EQ-5D-5 L and SF-12 was evaluated by first, examining the extent to which each instrument distinguished between participants with or without arthritis, and then, among those with arthritis, based on the level of pain (no, mild, moderate, severe) as a proxy for severity of the condition. The number of other chronic conditions (none, 1, 2 or 3+ chronic conditions), and self-reported health (excellent, very good, good and fair/poor) were used as indicators of the overall health of participants. One-way ANOVA, independent group t-test and chi-square test were used to test differences between examined groups as appropriate. Effect size was used to examine the magnitude of differences and was calculated as the difference in mean scores between participants with and those without arthritis, divided by the pooled standard deviation. Effect size was interpreted as: 0.2–0.49 small; 0.5–0.79 moderate and > =0.8 as large [19].

We hypothesized that EQ-5D-5 L index score, VAS, SF-12 domain and summary scores and SF-6D index score would all be lower in arthritis patients with higher levels of pain severity, while an inverse relationship was expected for the EQ-5D-5 L dimensions. Secondly, EQ-5D-5 L index score, VAS, SF-12 domain and summary scores and SF-6D index score were expected to be lower in arthritis patients with more comorbidities, with an inverse relationship for EQ-5D-5 L dimensions. Finally, EQ-5D-5 L index score, VAS, SF-12 domain and summary scores and SF-6D index score were expected to be lower in arthritis patients with poorer general health status, with an inverse relationship for EQ-5D-5 L dimensions.

The relationships between the EQ-5D-5 L and the SF-12 components were examined using Spearman’s correlation coefficients. Coefficients of < 0.20 were considered absent, 0.20–0.34 as weak, 0.35–0.50 as moderate and > 0.5 was considered strong correlation [20]. We expected mobility, self-care, usual activities and pain/discomfort dimensions of the EQ-5D-5 L to have a strong negative correlation with the SF-12 PCS and PF, RF, BP, and GH. Anxiety/depression dimension of the EQ-5D-5 L was also expected to have a strong negative correlation with the SF-12 MCS, SF, RE, and MH. A negative weak correlation was expected between the mobility, self-care, usual activities and pain/discomfort dimensions of the EQ-5D-5 L and mental health domains and summary scores (MCS, SF, RE, and MH) of the SF-12.

## Results

### General characteristics of participants

Of the 6275 respondents to the ARTA survey, 2844 had complete EQ-5D-5 L and SF-12 data and were included in this analysis. The average age of participants was 68.6 (SD 5.9; range 51 to 86) years; 55% were female. Half (51%) of the participants had a university education, 91% were retired, 41% had total annual household income between CAD$60,000-99,999, and 97% were Caucasian (Table 1). Participants had an average BMI of 27.2 (SD 4.8; range 13.1 to 56.8), with 64% being overweight or obese. Participants reported an average of 2.0 (SD 1.7) chronic conditions, while 34% reported 3 or more chronic conditions. The most common chronic condition among this population was arthritis (37%). Other musculoskeletal conditions, including osteoporosis, fibromyalgia and back problems, were reported by 31%, while high lipids and eye diseases were reported by 23 and 24%, respectively (Table 1).

**Table 1:** General characteristics of participants; overall and by the presence of arthritis

	Overall (*N* = 2844)	Without Arthritis (*N* = 1802)	With Arthritis (*N* = 1042)	*P*-value
	Mean ± SD, or N(%)	Mean ± SD, or N(%)	Mean ± SD, or N(%)	
Age – years	68.6 ± 5.9	68.1 ± 5.8	69.4 ± 5.9	< 0.001
50–64	520 (22.3)	360 (25.4)	160 (17.5)	
65–74	1442 (61.7)	862 (60.8)	580 (63.3)	
> = 75	373 (16.0)	197 (13.9)	176 (19.2)	
Gender				
Male	1286 (45.2)	883 (49.0)	403 (38.7)	< 0.001
Female	1558 (54.8)	919 (51.0)	639 (61.3)	
Highest level of Education				
Some high School/College	305 (10.7)	188 (10.4)	117 (11.2)	0.800
University/college	1450 (51.0)	923 (51.2)	527 (50.6)	
Graduate	1089 (38.3)	691 (38.4)	398 (38.2)	
Current employment status				
Retired	2595 (91.2)	1625 (90.2)	970 (93.1)	0.047
Full-time employee	33 (1.2)	23 (1.3)	10 (1.0)	
Part-time employee	140 (4.9)	103 (5.7)	37 (3.6)	
Other	76 (2.7)	51 (2.8)	25 (2.4)	
Total annual household income				
< $60,000	787 (27.7)	479 (26.6)	308 (29.6)	< 0.001
$60,000–$99,999	1183 (41.6)	749 (41.6)	434 (41.7)	
> $100,000	473 (16.6)	335 (18.6)	138 (13.2)	
Prefer not to answer	401 (14.1)	239 (13.3)	162(15.6)	
Ethnicity				
White	2747 (96.6)	1735 (96.3)	1012 (97.1)	0.235
Non-White	97 (3.4)	67 (3.7)	30 (2.9)	
BMI	27.2 ± 4.8	26.8 ± 4.6	27.8 ± 5.2	< 0.001
Total No. of chronic conditions (0–13)	2.02 ± 1.70	1.35 ± 1.37	2.17 ± 1.59	< 0.001
No chronic condition	610 (21.5)	610 (33.8)	144 (13.8)	< 0.001
1 chronic condition	638 (22.4)	494 (27.4)	255 (24.5)	
2 chronic conditions	617 (21.7)	362 (20.1)	258 (24.8)	
3+ chronic conditions	979 (34.4)	336 (18.7)	385 (36.9)	

*BMI* body mass index

The mean EQ-5D-5 L index score was 0.86 (SD 0.11), with a score ranging from 0 to 0.95, and a distribution skewed towards full health with a ceiling effect of 23% (*n* = 662) (Additional file 1: Figure S1). The mean SF-6D index score was 0.79 (0.13), with a score ranging from 0.37 to 1, and a bi-modal distribution with a ceiling effect of 7% (*n* = 194) (Additional file 1: Figure S1). For respondents with arthritis, the mean age was 68.1 (SD 5.8) years, and 61% were female (Table 1). The respondents with arthritis also reported more comorbidities, with 37% reporting 3 or more other chronic conditions compared to 18.7% in those without arthritis.

### The relationship between EQ-5D-5 L and SF-12

EQ-5D-5 L and SF-6D index scores had a strong and positive correlation (r = 0.63) (Additional file 1: Table S1). There was a strong negative correlation between related dimensions such as mobility-PF (r = − 0.60), usual activity-RP (r = − 0.61), pain/discomfort-BP (r = − 0.70) and anxiety/depression-MH (r = − 0.60). PCS was negatively and strongly correlated with the EQ-5D-5 L mobility, usual activities, and pain dimensions (r = − 0.60 to − 0.65). MCS also had a strong negative correlation with anxiety/depression (r = − 0.59). As expected, mobility and pain/discomfort had weak to no correlation with RE and MH domains (r = 0.20 to − 0.34) (Additional file 1: Table S1).

### Discriminative validity of EQ-5D-5 L and SF-12

The mean difference in index scores between participants with and without arthritis was 0.05 for EQ-5D-5 L and 0.06 for SF-6D, representing a moderate effect size (0.5) for both index scores (Table 2). The difference between the number of participants with and without arthritis who reported having problems on the EQ-5D-5 L pain/discomfort and mobility dimensions was 25 and 28%, respectively. However, the differences between those with and without arthritis who reported problems with self-care and feeling anxious/depressed were only 4 and 7%, respectively. Participants with arthritis also had lower PCS (by 4.4) and MCS (by 2.4) scores compared to those without arthritis (Table 2). Effect sizes were moderate for PCS (0.5), PF (0.5), RP (0.5), while BP had the largest effect size of 0.6 (Table 2).

**Table 2:** EQ-5D-5 L dimensions, index and VAS scores and SF-12 domains, composite scores and SF-6D; overall and by presence of arthritis

	Overall (*N* = 2844) Mean ± SD, or N(%)	Without Arthritis (*N* = 1802) Mean ± SD, or N(%)	With Arthritis (*N* = 1042) Mean ± SD, or N(%)		
Mobility					
level 1	1957 (68.8)	1429 (79.3)	528 (50.7)		
level 2	582 (20.5)	262 (14.5)	320 (30.7)		
levels 3, 4, 5	305 (10.7)	111 (6.2)	194 (18.6)		
Self-care					
level 1	2718 (95.6)	1751 (97.2)	967 (92.8)		
level 2	79 (2.8)	28 (1.6)	51 (4.9)		
level 3, 4, 5	47 (1.6)	23 (1.3)	24 (2.3)		
Usual Activities					
level 1	2104 (74.0)	1486 (82.5)	618 (59.3)		
level 2	532 (18.7)	230 (12.8)	302 (29.0)		
level 3, 4, 5	208 (7.3)	86 (4.8)	122 (11.7)		
Pain/Discomfort					
level 1	848 (29.8)	710 (39.4)	138 (13.2)		
level 2	1557 (54.8)	926 (51.4)	631 (60.6)		
level 3, 4, 5	439 (15.4)	166 (9.2)	273 (26.2)		
Anxiety/ Depression					
level 1	2046 (71.9)	1343 (74.5)	703 (67.5)		
level 2	667 (23.5)	397 (22.1)	270 (25.9)		
level 3, 4, 5	131 (4.6)	62 (3.4)	69 (6.6)		
				Difference	Effect size
EQ-5D-5 LIndex score	0.86 ± 0.11	0.88 ± 0.09	0.83 ± 0.13	0.05	0.5
EQ-VAS	81.9 ± 14.1	83.3 ± 13.8	79.5 ± 14.3	3.8	0.3
PF	49.7 ± 9.2	51.2 ± 8.4	47.0 ± 9.9	4.2	0.5
RP	48.8 ± 8.9	50.3 ± 8.4	46.2 ± 9.0	4.1	0.5
BP	49.4 ± 8.7	51.4 ± 7.7	46.1 ± 9.1	5.3	0.6
GH	52.6 ± 8.2	53.6 ± 8.0	50.9 ± 8.3	2.6	0.3
VT	52.7 ± 7.7	53.8 ± 7.4	50.9 ± 8.0	2.9	0.4
SF	51.7 ± 7.9	52.7 ± 7.1	50.1 ± 8.9	2.5	0.3
RE	49.9 ± 9.2	50.8 ± 8.6	48.4 ± 10.1	2.3	0.3
MH	53.7 ± 7.4	50.3 ± 7.1	52.7 ± 7.7	1.6	0.2
SF-6D	0.79 ± 0.13	0.81 ± 0.12	0.75 ± 1.3	0.06	0.5
PCS	49.7 ± 8.4	51.3 ± 7.7	46.9 ± 8.7	4.4	0.5
MCS	52.8 ± 7.0	53.7 ± 6.5	51.3 ± 7.5	2.4	0.3

All differences were statistically significant at *P* < 0.001

Both EQ-5D-5 L and SF-6D index scores were significantly different across the groups defined by severity of pain, number of comorbidities and self-reported general health (Tables 3, 4 and 5), with differences ranging from 0.02 to 0.21 for EQ-5D-5 L index scores, and 0.02 to 0.13 for SF-6D index scores. Participants with more comorbidities had lower EQ-5D-5 L index scores, and more problems on the EQ-5D-5 L dimensions compared to those with fewer comorbidities. Similarly, those with lower self-rating of health had lower EQ-5D-5 L index scores and more problems on each of the dimensions. The SF-12 domain and summary scores were lower for participants with higher levels of chronic pain, more comorbidities, and lower general self-rated health status.

**Table 3:** EQ-5D-5 L dimensions, VAS and index scores and SF-12 domains, composite scores and SF-6D by chronic pain for individuals with arthritis (*n* = 1042)

	No Pain *N* = 17 (1.6) Mean ± SD, or N(%)	Mild Pain *N* = 531 (51.1) Mean ± SD, or N(%)	Moderate Pain *N* = 309 (29.8) Mean ± SD, or N(%)	Severe Pain 182 (17.5) Mean ± SD, or N(%)
Mobility				
level 1	12 (70.6)	298 (56.1)	121 (39.2)	96 (52.8)
level 2	5 (29.4)	178 (33.5)	94 (30.4)	41 (22.5)
level 3,4,5	0 (0)	55 (10.4)	94 (30.4)	45 (24.7)
Self-care				
level 1	17 (100)	512 (96.4)	278 (90.0)	157 (86.3)
level 2	0 (0)	16 (3.0)	20 (6.5)	15 (8.2)
level 3,4,5	0 (0)	3 (0.6)	11 (3.5)	10 (5.5)
Usual activities				
level 1	15 (88.2)	361 (68.0)	137 (44.3)	103 (56.6)
level 2	2 (11.8)	143 (26.9)	113 (36.6)	43 (23.6)
level 3,4,5	0 (0)	27 (5.1)	59 (19.1)	36 (19.8)
Pain/Discomfort				
level 1	11 (64.7)	70 (13.2)	22 (7.1)	35 (19.2)
level 2	6 (35.3)	395 (74.4)	143 (46.3)	84 (46.2)
level 3,4,5	0 (0)	66 (12.4)	144 (46.6)	63 (34.6)
Anxiety/Depression				
level 1	16 (94.1)	387 (72.9)	175 (56.6)	123 (67.6)
level 2	1 (5.9)	125 (23.5)	99 (32.01)	44 (24.2)
level 3,4,5	0 (0)	19 (3.6)	35 (11.3)	15 (8.2)
Index score	0.92 ± 0.04	0.86 ± 0.08	0.79 ± 0.13	0.79 ± 0.19
VAS score	85.5 ± 7.8	81.6 ± 11.7	76.3 + 16.6	78.0 ± 15.8
PF	50.9 ± 6.1	48.9 ± 8.7	44.3 ± 10.7	46.0 ± 11.1
RP	51.6 ± 8.1	47.6 ± 7.9	43.9 ± 9.4	45.3 ± 10.6
BP	53.4 ± 6.7	48.4 ± 6.8	43.2 ± 9.4	43.6 ± 12.0
GH	55.6 ± 3.4	52.2 ± 7.3	49.3 ± 8.6	49.7 ± 10.1
VT	54.1 ± 9.6	52.0 ± 7.0	49.1 ± 8.2	50.0 ± 9.4
SF	55.0 ± 3.4	51.7 ± 7.7	47.7 ± 9.7	49.1 ± 10.7
RE	53.5 ± 7.1	50.2 ± 8.6	46.2 ± 10.9	46.7 ± 12.0
MH	54.2 ± 9.0	53.9 ± 6.8	50.6 ± 8.5	52.7 ± 7.9
SF-6D	0.85 ± 0.11	0.78 ± 0.11	0.71 ± 0.12	0.74 ± 0.15
PCS	52.6 ± 6.1	48.7 ± 7.0	44.2 ± 9.2	45.5 ± 11.0
MCS	54.7 ± 6.6	52.8 ± 6.4	48.9 ± 8.1	50.6 ± 8.8

All differences were statistically significant at *P* < 0.001

**Table 4:** EQ-5D-5 L dimensions, VAS, index scores and SF-12 by Comorbidities for individuals with arthritis

	No comorbidity *N* = 144 (13.8) Mean ± SD, or N(%)	1 comorbidity *N* = 255 (24.5) Mean ± SD, or N(%)	2 comorbidities *N* = 258 (24.8) Mean ± SD, or N(%)	3+ comorbidities *N* = 385 (36.9) Mean ± SD, or N(%)
Mobility				
level 1	93 (64.6)	133 (52.1)	145 (56.2)	157 (40.8)
level 2	35 (24.3)	93 (36.5)	67 (26.0)	125 (32.4)
level 3,4,5	16 (11.1)	29 (11.4)	46 (17.8)	103 (26.8)
Self-care				
level 1	139 (96.5)	249 (97.6)	240 (93.0)	339 (88.1)
level 2	4 (2.8)	5 (2.0)	13 (5.0)	29 (7.5)
level 3,4,5	1 (0.7)	1 (0.4)	5 (2.0)	17 (4.4)
Usual activities				
level 1	109 (75.7)	175 (68.6)	160 (62.0)	174 (45.2)
level 2	33 (22.9)	65 (25.5)	64 (24.8)	140 (36.4)
level 3,4,5	2 (1.4)	15 (5.9)	34 (13.2)	71 (18.4)
Pain/Discomfort				
level 1	28 (19.4)	47 (18.4)	38 (14.7)	25 (6.5)
level 2	95 (66.0)	162 (63.5)	146 (56.6)	228 (59.2)
level 3,4,5	21 (14.6)	46 (18.1)	74 (28.7)	132 (34.3)
Anxiety/Depression				
level 1	113 (78.5)	190 (74.5)	176 (68.2)	224 (58.2)
level 2	28 (19.4)	55 (21.6)	67 (26.0)	120 (31.2)
level 3,4,5	3 (2.1)	10 (3.9)	15 (5.8)	41 (10.6)
Index score	0.87 ± 0.07	0.85 ± 0.09	0.83 ± 0.13	0.79 ± 0.15
VAS score	85.4 ± 9.7	82.6 ± 12.2	79.4 ± 14.0	75.2 ± 15.8
PF	50.5 ± 8.3	49.7 ± 8.1	46.9 ± 10.1	44.1 ± 10.7
RP	50.1 ± 7.8	48.1 ± 8.1	46.4 ± 8.9	43.3 ± 9.3
BP	48.7 ± 8.0	48.5 ± 7.6	46.0 ± 9.7	43.6 ± 9.3
GH	54.8 ± 5.7	52.7 ± 7.2	51.0 ± 8.4	48.2 ± 9.0
VT	53.8 ± 6.7	52.7 ± 6.8	50.6 ± 8.3	48.7 ± 8.3
SF	52.9 ± 7.1	51.9 ± 7.4	50.4 ± 8.9	47.8 ± 10.0
RE	51.4 ± 7.9	50.1 ± 8.8	48.8 ± 9.4	46.0 ± 11.5
MH	54.0 ± 6.7	54.1 ± 7.1	52.8 ± 7.4	51.3 ± 8.4
SF-6D	0.80 ± 0.12	0.78 ± 0.12	0.75 ± 0.13	0.72 ± 0.12
PCS	50.8 ± 6.8	49.2 ± 7.0	47.0 ± 8.9	43.7 ± 9.2
MCS	53.7 ± 5.9	53.0 ± 6.6	51.4 ± 7.3	49.2 ± 8.4

All differences were statistically significant at *P* < 0.001

**Table 5:** EQ-5D-5 L dimensions, VAS, index scores and SF-12 by General Health status for individuals with arthritis

	Excellent *N* = 123 (11.8) Mean ± SD, or N(%)	Very good *N* = 506 (48.5) Mean ± SD, or N(%)	Good *N* = 333 (32.0) Mean ± SD, or N(%)	Fair/Poor *N* = 80 (7.7) Mean ± SD, or N(%)
Mobility				
level 1	98 (79.7)	303 (59.9)	122 (36.6)	5 (6.3)
level 2	18 (14.6)	163 (32.2)	122 (36.6)	17 (21.2)
level 3,4,5	7 (5.7)	40 (7.9)	89 (26.8)	52 (72.5)
Self-care				
level 1	122 (99.2)	495 (97.8)	307 (92.2)	43 (53.8)
level 2	0 (0)	10 (2.0)	18 (5.4)	23 (28.7)
level 3,4,5	1 (0.8)	1 (0.2)	8 (2.4)	14 (17.5)
Usual activities				
level 1	104 (84.6)	363 (71.7)	144 (43.3)	7 (8.8)
level 2	14 (11.4)	128 (25.3)	139 (41.7)	21 (26.2)
level 3,4,5	5 (4.0)	15 (3.0)	50 (15.0)	46 (65.0)
Pain/Discomfort				
level 1	34 (27.6)	86 (17.0)	18 (5.4)	0 (0)
level 2	81 (65.9)	349 (69.0)	184 (55.3)	17 (21.3)
level 3,4,5	8 (6.5)	71 (14.0)	131 (39.3)	63 (78.7)
Anxiety/Depression				
level 1	100 (81.3)	381 (75.3)	203 (61.0)	19 (23.8)
level 2	21 (17.1)	111 (21.9)	104 (31.2)	34 (45.5)
level 3,4,5	2 (1.6)	14 (2.8)	26 (7.8)	21 (33.7)
Index score	0.89 ± 0.07	0.87 ± 0.06	0.80 ± 0.11	0.59 ± 0.21
VAS score	92.0 ± 8.0	84.9 ± 8.5	73.0 ± 11.4	52.5 ± 15.5
PF	53.1 ± 6.5	50.2 ± 7.4	43.4 ± 9.8	33.0 ± 10.0
RP	52.2 ± 6.3	49.1 ± 7.3	42.8 ± 7.9	32.5 ± 8.0
BP	51.9 ± 6.4	48.8 ± 7.0	42.89 ± 8.7	33.5 ± 9.5
GH	61.7 ± 0	55.5 ± 0	45.1 ± 0	29.8 ± 2.8
VT	56.6 ± 6.1	53.1 ± 5.9	48.3 ± 7.4	38.2 ± 7.8
SF	53.8 ± 6.4	52.7 ± 6.2	48.0 ± 9.3	37.0 ± 11.2
RE	52.4 ± 6.2	51.0 ± 8.0	46.8 ± 10.0	33.4 ± 12.5
MH	54.9 ± 7.1	54.5 ± 6.5	51.3 ± 7.6	43.9 ± 8.5
SF-6D	0.83 ± 0.12	0.79 ± 0.10	0.71 ± 0.11	0.58 ± 0.09
PCS	53.8 ± 4.9	50.3 ± 5.8	43.0 ± 7.2	30.8 ± 7.8
MCS	55.2 ± 5.6	53.8 ± 5.4	49.2 ± 6.9	38.4 ± 8.3

All differences were statistically significant at *P* < 0.001

Participants with moderate-severe chronic pain on the pain rating scale reported similar mean EQ-5D-5 L index scores (0.79, SD 0.13 and 0.79, SD 0.19 respectively). Contrary to expectations, participants with moderate chronic pain on the pain rating scale reported lower SF-6D index scores (0.71, SD 0.12) compared to those with severe chronic pain (0.74, SD 0.15). The same reverse pattern was also observed in the EQ-5D-5 L dimensions and SF-12 domain and summary scales (Table 3). Counter to our expectations, 19% of participants with severe chronic pain reported no problems on the pain/discomfort dimension of the EQ-5D-5 L.

## Discussion

In this study, we found that the EQ-5D-5 L and SF-6D index scores demonstrated moderate discriminative validity in this population. However, the EQ-5D-5 L dimensions and SF-12 domain scores demonstrated limited discriminative validity with small effect sizes. This was consistent with previous reports on the EQ-5D-5 L and SF-36, SF-12 and SF-6D in the general population, people with chronic pain, osteoarthritis and type 2 diabetes [21–28]. Significant differences in the discriminative validity of the EQ-5D-5 L dimensions and the SF-12 domains have also been reported in literature; however, the magnitudes of the differences were small to moderate [21–23]. Furthermore, more participants responded to levels 3,4 and 5 of the EQ-5D-5 L compared to the responses on the level 3 for the EQ-5D-3 L as reported in literature [5]. The difference could be attributed to the high ceiling effect of the earlier 3 L version of the EQ-5D. The use of Canadian value set for the EQ-5D-5 L index score and UK value set for the SF-6D index score could also contribute to the differences observed in this study [23, 24, 26].

An important observation in this study was the limited ability of both the EQ-5D-5 L and SF-12 to discriminate between participants with moderate versus severe chronic pain of 6-months duration. Participants with moderate chronic pain reported more problems on the EQ-5D-5 L pain/discomfort dimension compared to those with severe chronic pain. In addition, 19% of participants who reported severe pain on the chronic pain scale over the last 6 months, reported no problems on the pain/discomfort dimension of the EQ-5D. These results could be related to the reference period for reporting the pain level in each of these instruments. The EQ-5D-5 L asks respondents to indicate their level of pain/discomfort “*today*”, the SF-12 assesses pain “*over the past 4 weeks*”, while the chronic pain scale asks about pain over the *last 6 months*. Stone et al. [29] employed a novel Ecological Momentary Assessment (EMA) approach to assess pain over a week in patients with rheumatoid arthritis. They found large individual differences in variation of pain and fatigue over time and at different times of the day. In our current study, it is possible that participants reporting severe pain of 6 months duration on the chronic pain scale could have been experiencing very mild to moderate pain on the day (based on the EQ-5D-5 L) or during last 4 weeks (based on the SF-12) of assessment of the pain. Momentary assessment of pain in our study could have identified times within the day in which participants had higher or lower levels of pain, which could be attributed to medication intake, stressors or adequacy of sleep [29].

This study has some limitations. Firstly, the study had a low response rate and was limited to well educated, Caucasian, older adults, which is to a great extent inherent in the fact that the sampling frame was retired teachers and their dependents. This limits the generalizability of these results to other arthritis populations that may be different from this sample. Second, the Canadian value set was used for calculating the EQ-5D-5 L index score, while the UK value set was used for the SF-6D index score. It is possible that the UK general population has different valuations of health from the Canadian general population. However, this only affects the index scores and not the dimensions or domains of the instruments. Third, self-reported data has the potential of being under or over reported, which could bias our results. We are unable to estimate the extent to which this may have happened in this survey; nonetheless, even if the prevalence of arthritis may have been under-reported, this would not impact the assessment of discriminative validity of these instruments. Finally, we did not have data on the type, location, and severity of arthritis or the pain intensity; this information would have been very useful in examining the discriminative validity of these instruments in patient sub-groups and should be explored in future research.

## Conclusion

This study found moderate discriminative validity for the EQ-5D-5 L and SF-6D index scores. Pain/discomfort and mobility dimension of the EQ-5D-5 L and bodily pain domain of the SF-12 demonstrated the highest discriminative power. However, the results suggest that these instruments are limited in their ability to capture the burden of pain in this patient population due to variation of pain duration and severity over time. This has an important implication for researchers and clinicians who use these tools in the assessment of chronic pain. The duration of pain and the time of assessment of pain are very important in capturing HRQL; these should be considered in interpreting data obtained from these instruments in patients with arthritis.

## Additional file

Additional file 1: **Table S1.** Correlation Matrix for EQ-5D-5 L and SF-12 (Overall sample). **Figure S1.** Scatter Plot of EQ-5D-5 L index Score and SF-6D index score. (DOCX 472 kb)

## Abbreviations

ARTA: Alberta Retired Teachers Association
BMI: Body Mass Index
BP: Bodily Pain
EMA: Ecological Momentary Assessment
EQ-5D-5 L: EuroQol 5 Dimensions 5 Levels
HRQL: Health-Related Quality of Life
MCS: Mental Component Summary
MH: Mental Health
MID: Minimally Important Difference
PCS: Physical Component Summary
PF: Physical Functioning
RP: Role Physical
SF: Social Functioning
SF-12: Short Form Survey 12 Questions
SF-36: Short Form Survey 36 Questions
SF-6D: Short Form Survey 6 Dimensions
VAS: Visual Analogue Scale
VT: Vitality
WOMAC: Western Ontario and McMaster Universities Osteoarthritis Index
