# Supplementary material for: Discriminative validity of the EQ-5D-5 L and SF-12 in older adults with arthritis
Source: Health Qual Life Outcomes. 2019 Apr 17;17:68. doi: 10.1186/s12955-019-1129-6 (PMC6469074; doi:10.1186/s12955-019-1129-6)
Supplement: Supplementary file 1 — Table S1. Correlation Matrix for EQ-5D-5 L and SF-12 (Overall sample). Figure S1. Scatter Plot of EQ-5D-5 L index Score and SF-6D index score. (DOCX 472 kb) [file 12955_2019_1129_MOESM1_ESM.docx]

Table S1: Correlation Matrix for EQ-5D-5L and SF-12 (Overall sample)

|  | Mobility | Self-care | Usual activities | Pain/ Discomfort | Anxiety/ Depression | EQ-5D Index | EQ-5D VAS |
| --- | --- | --- | --- | --- | --- | --- | --- |
| **SF-6D** | -0.47 | -0.29 | **-0.56** | **-0.5** | **-0.5** | **0.63** | **0.52** |
| PCS | **-0.61** | -0.31 | **-0.65** | **-0.6** | -0.31 | **0.62** | **0.72** |
| MCS | -0.36 | -0.26 | -0.46 | -0.43 | **-0.59** | **0.5** | **0.57** |
| PF | **-0.6** | -0.31 | **-0.59** | -0.45 | -0.2 | 0.46 | 0.57 |
| RP | **-0.55** | -0.3 | **-0.61** | **-0.51** | -0.28 | **0.53** | 0.62 |
| BP | **-0.56** | -0.3 | **-0.58** | **-0.7** | -0.25 | 0.48 | **0.71** |
| GH | -0.43 | -0.23 | -0.45 | -0.45 | -0.26 | **0.74** | 0.52 |
| VT | -0.41 | -0.27 | -0.49 | -0.42 | -0.36 | **0.53** | 0.52 |
| SF | -0.41 | -0.32 | **-0.52** | -0.41 | -0.43 | 0.42 | **0.52** |
| RE | -0.32 | -0.26 | -0.43 | -0.34 | -0.47 | 0.37 | 0.47 |
| MH | -0.2 | -0.19 | -0.28 | -0.28 | **-0.6** | 0.33 | 0.4 |

Bolded values indicate strong correlation (≥ 0.50).

PCS-Physical Component Score, MCS-Mental Component summary score, PF-Physical Function, RP- Role Physical, BP-Bodily Pain, GH-General Health, VT-Vitality, SF-Social functioning, RE-Role Emotional and MH-Mental Health.

Figure S1: Scatter Plot of EQ-5D-5L index Score and SF-6D index score.
